# Extraordinary variability in gene activation and repression programs during gonadal sex differentiation across vertebrates

**DOI:** 10.3389/fcell.2024.1328365

**Published:** 2024-01-23

**Authors:** Núria Sánchez-Baizán, Ignasi Jarne-Sanz, Álvaro S. Roco, Manfred Schartl, Francesc Piferrer

**Affiliations:** ^1^ Institut de Ciències del Mar (ICM), Spanish National Research Council (CSIC), Barcelona, Spain; ^2^ Developmental Biochemistry, Biocenter, University of Wuerzburg, Wuerzburg, Germany; ^3^ Department of Experimental Biology, Faculty of Experimental Sciences, University of Jaén, Jaén, Spain; ^4^ Xiphophorus Genetic Stock Center, Texas State University, San Marcos, TX, United States

**Keywords:** gonadal development, gene activation, gene repression, gene expression networks, transcriptomics, sex determination, sex differentiation, sex markers

## Abstract

Genes involved in gonadal sex differentiation have been traditionally thought to be fairly conserved across vertebrates, but this has been lately questioned. Here, we performed the first comparative analysis of gonadal transcriptomes across vertebrates, from fish to mammals. Our results unambiguously show an extraordinary overall variability in gene activation and repression programs without a phylogenetic pattern. During sex differentiation, genes such as *dmrt1*, *sox9*, *amh*, *cyp19a* and *foxl2* were consistently either male- or female-enriched across species while many genes with the greatest expression change within each sex were not. We also found that downregulation in the opposite sex, which had only been quantified in the mouse model, was also prominent in the rest of vertebrates. Finally, we report 16 novel conserved markers (e.g., *fshr* and *dazl*) and 11 signaling pathways. We propose viewing vertebrate gonadal sex differentiation as a hierarchical network, with conserved hub genes such as *sox9* and *amh* alongside less connected and less conserved nodes. This proposed framework implies that evolutionary pressures may impact genes based on their level of connectivity.

## 1 Introduction

Sexual systems in vertebrates are diverse and include gonochorism (or separate sexes), hermaphroditism and unisexuality (Pla et al., 2022). During development, the fate of the bi-potential gonad is first established by the process of sex determination, followed by the process of gonadal sex differentiation, in which it is transformed into ovaries or testis. Sex-determining systems in vertebrates range from environmental (ESD) to genotypic (GSD), depending on the factors or combination of them that trigger the decision. Today GSD and ESD are seen as two ends of a continuum rather than two mutually exclusive options (Sarre et al., 2004; Heule et al., 2014).

The genes involved in sex differentiation are fairly conserved from fish to mammals (Piferrer and Guiguen, 2008; Rhen and Schroeder, 2017; Hirst et al., 2018; Yildirim et al., 2020; Nagahama et al., 2021; Ruiz-García et al., 2021; Stöck et al., 2021). However, most transcriptomic studies carried out so far have focused on sex-specific differences in gene expression (and less frequently with the associated gene networks) at a particular developmental stage (Supplementary Figure S1A). Using this approach, the consensus emerged that genes involved were relatively conserved during vertebrate sex differentiation. Yet, recently, it has become apparent that the temporal and relative expression of some key genes, as well as the relative position of these genes within the network, are different across vertebrates. In some instances, there are genes whose function is even related to the opposite sex (Nagahama et al., 2021). For example, in mouse, testis development is determined by the expression of the Sex Determining Region Y (*Sry*) gene, which activates SRY-Box Transcription Factor 9 (*Sox9*) (Rahmoun et al., 2017). In turn, *Sox9* activates a positive feedback loop between fibroblast growth factor 9 (*Fgf9*) and lipocalin type prostaglandin D2 synthase (*Ptgds*) (Colvin et al., 2001; Moniot et al., 2009). Anti-Müllerian hormone (*Amh*) and Doublesex And Mab-3 Related Transcription Factor 1 (*Dmrt1*) is another target of *Sox9*, and its expression in Sertoli cells causes the regression of Müllerian ducts between 13.5 and 14.5 days post coitum (dpc) (Behringer et al., 1994; Nagahama et al., 2021). Nevertheless, in chicken, testis differentiation is initiated by *DMRT1* and activates *AMH* and Hemogen (*HEMGN*). In turn, those genes lead to the expression of *SOX9* (instead of *Sox9* being followed by *Amh* and *Dmrt1* in the mouse) (Nagahama et al., 2021). This, therefore, constitutes an example of genes being expressed in different order.

One approach to investigate sexual differentiation in a manner different from the male vs. female approach mentioned above is to analyze gene expression dynamics within each sex but at different developmental stages during sex differentiation (Supplementary Figure S1B). Munger et al. (2013) used this approach to study transcriptomic dynamics during gonadal sex differentiation in the mouse. Importantly, such approach revealed that sexually dimorphic gene expression patterns are established not only by activation but also by repression programs (Munger et al., 2013; Capel, 2017). Moreover, they combined the fold change (FC) expression values between developmental stages for each sex (i.e., two FC values) into a single data point using Cartesian coordinates. This allowed quantifying the proportion of genes that are regulated by each one of the following three types of mechanisms: 1) genes that are upregulated (activated) in one sex with concomitant downregulation (repression) in the opposite sex, 2) genes that are upregulated in one sex with constant expression in the opposite sex, or 3) genes that exhibit constant expression in one sex with combined active downregulation in the opposite sex.

Surprisingly, and to the best of our knowledge, such approach to visualize gene expression dynamics within each sex at different developmental stages during sex differentiation has never been used again in the 10 years elapsed since the seminal study of Munger et al. (2013). Such a two-stage comparison approach accurately characterized overall changes in gene expression. It revealed that testis development required the upregulation of most male-related genes with concomitant downregulation of a considerable number of female-related genes. In contrast, ovarian differentiation involved the upregulation of a lower number of female-related genes and downregulation of a very small amount of male-related genes. Thus, the type of gene expression dynamics that were described for the mouse using this approach remain essentially undescribed for the rest of the vertebrate species where sex differentiation has been studied. Analysis of gene expression dynamics is an important tool for studying position within the network, temporal and relative expression of genes involved in complex developmental processes (Reis et al., 2001; Batut et al., 2022).

Here, we used both published and unpublished data to further exploit this approach in other vertebrate species. We selected species for which the transcriptome was available at the beginning (here referred as T1) and towards the end (referred as T2) of gonadal sex differentiation, representing five of the major groups of vertebrates: the European sea bass (*Dicentrarchus labrax*) (Ribas et al., 2019), the platyfish (*Xiphophorus maculatus*), the clawed frog (*Xenopus laevis*) (Piprek et al., 2018), the red-eared slider turtle (*Trachemys scripta elegans*) (Czerwinski et al., 2016), and the chicken (*Gallus gallus*) (Ayers et al., 2013). Of note, we also included the mouse (*Mus musculus*) not only as a representative of mammals but also to replicate previous results (Munger et al., 2009; Munger et al., 2013).

First, genes that become enriched in males relative to females at T2 were named Male-enriched genes (MEGs), while genes that become enriched in females relative to males at T2 were named Female-enriched genes (FEGs). Second, we analyzed gene expression dynamics to calculate the expression FC between T1 and T2 for each sex separately. Additionally, we identified not only which genes were relevant for gonadal sex differentiation, but also their dynamics in both sexes and compared them across vertebrates, i.e., we quantified the number of genes that change their expression between T1 and T2 through each of the three mechanisms mentioned above. Furthermore, we identified the genes with the highest expression change between stages and determined whether those were conserved or not across species. This allowed the identification of new potential markers of early sex differentiation in vertebrates. Lastly, we identified the most conserved pathways enriched by FEGs and MEGs across vertebrates.

## 2 Materials and methods

### 2.1 Species selection and data collection

We carefully selected comparable gonadal transcriptome datasets (microarray or RNA-seq) for each of the five vertebrate groups during sex differentiation. The selection criteria included the sex determining system, two developmental stages, i.e., at the beginning of sexual differentiation (T1), when gonads were still morphologically undifferentiated; and towards the end of sex differentiation (T2), and samples availablility. When data from several stages was available, careful examination of the development dynamics was made in order to determine which ones were most equivalent (Roux et al., 2015). A minimum of two samples per sex and stage were included (i.e., eight or more samples per species) for statistical purposes. The species selected were European sea bass (*D. labrax*), platyfish (*X. maculatus*), African clawed frog (*X. laevis*), red-eared slider turtle (*T. scripta elegans*), chicken (*G. gallus*) and mouse (*M. musculus*). Details on selected species, developmental stages, source of data and data format are summarized in Supplementary Table S1, and details on the background biology of the selected species can be found in the Supplementary Materials and methods section.

#### 2.1.1 Fishes

To represent fish, gonadal transcriptome data of the sea bass and the platyfish were used. To work with the gonadal transcriptome of the sea bass, we selected samples of four females and seven males at 110 days post fertilization (dpf) when the gonads are morphologically undifferentiated but can be sexed by gene expression of sex markers (i.e., *cyp19a1a*, (Blázquez et al., 2009)), and twelve gonads (six testes and six ovaries) of fish at 250 dpf, towards the end of differentiation (Ribas et al., 2019). Data are publicly accessible in GEO (accession number GSE11584)*.* The raw downloaded file consists of 43,801 probe copies representing 20,978 transcripts with quantile normalized expression values, and corrected for batch effect. To include another fish species with a different sex determining system we produced the gonadal transcriptome of the platyfish using RNA-sequencing technology.

#### 2.1.2 RNA sequencing of gonadal tissue during sex differentiation of the platyfish

The first stage selected for the platyfish was embryonic stage 24 and day 7 according to Tavolga (1949), which correspond to 17 dpf and 30 dpf, respectively. At stage 24 (17 dpf) the gonads are still morphologically undifferentiated but show already differential expression of the earliest gonadal male (*dmrt1*) and female (*cyp19a1a*) markers. The second stage, day 7 after birth (30 dpf), represents a later stage of testis and ovary development. Material from genotypic male and female individuals (10 for 17 dpf and 5 for 30 dpf) were pooled. RNA transcriptomes from two replicates of all samples were obtained from 100 bp paired-end Illumina (HiSeq 4,000) reads (approx. 20 million reads per sample). These data are available on the NCBI Sequence Read Archive (BioProject PRJNA944639).

#### 2.1.3 Amphibian, reptile, bird and mammal

To represent amphibians, we selected data from developing gonads of the African clawed frog published by Piprek et al. (2018) under accession number GSE105103. We used three samples for each sex at Niewkoop and Faber (NF) 50 and NF53, which produced a matrix with 20,219 annotated transcripts. In reptiles, we obtained transcriptomic data from Czerwinski et al. (2016), who studied the differentiating gonads of the red-eared slider turtle. For that species we used two replicates for developing testis and ovaries at stages 15 and 21. Data is available from the Sequence Read Archive (SRA) database under the code SRP079664. For birds, gonadal transcriptomic data of the chicken were obtained from Ayers et al. (2013). For the chicken, two replicates per sex were available at E4.5 and E6.0 stages. These data were downloaded from SRA under the accession number SRP014719. Finally, the gonadal transcriptome data of the mouse were obtained from Munger et al. (2009), and consisted of 25,697 annotated probes for three females and five males at E11.0 and three females and two males at E12.0. All the selected samples were from the strain 129S1, available from GEO with code GSE41948.

### 2.2 Data processing

The methods were implemented in R software and R studio (R Core Team, 2021; RStudio Team, 2021). The source code has been made publicly available on GitHub repository available from: https://github.com/Nsbaizan/Transcriptomic_dynamics.

On one hand, data of the European sea bass, the African clawed frog, and the mouse were obtained using microarrays. Therefore, normalized intensity datasets were downloaded from the National Center for Biotechnology Information (NCBI) database. On the other hand, data from the platyfish, the red-eared slider turtle and chicken were obtained with RNA-sequencing. Raw sequences of the red-eared slider turtle and chicken were downloaded from the European Nucleotide Archive (ENA) database. For such datasets, quality reports were produced using *FastQC* software (version 0.11.9) (Wingett and Andrews, 2018). Adapters and sequences with a quality score lower than 30 were trimmed with *Bbduk* software (version 38.90) (Bushnell, 2014). Reads were aligned against their respective reference genome using *Hisat2* software (version 2.2.1) (Kim et al., 2015). To map the platyfish reads the newest annotation available was used, the X_maculatus-5.0-male from 2021 in Ensembl, with 24,209 annotated genes. The red-eared slider turtle reads were mapped to CAS_Tse_1.0 genome acquired from NCBI which contains 28,415 annotated genes. The reference genome used to map chicken reads was GRCg6a from Ensembl, with a total of 20,937 genes. *Samtools* software (version 1.12) (Li et al., 2009) was used to convert and format aligned files and expression count matrices were obtained using *featureCounts* software (version 2.0.1) (Liao et al., 2014). Normalization, low gene expressed genes filter, and determination of differential expression were performed using DESeq2 package (v.1.34.0) (Love et al., 2014; Love et al., 2022). The genes kept for further analysis were those with FC ≥1.5 and *p*-value <0.05.

### 2.3 Determination of differentially expressed genes throughout sex differentiation

Determination of differentially expressed genes (DEGs) between T1 and T2 during sex differentiation was performed separately for each sex and each species. Specifically, the approach used to visualize the transcriptomic changes as described by Munger et al. (2013) consisted in plotting the significant FC expression between two developmental stages of genes with 1.5 log_2_ FC or higher expression in either sex between T1 and T2. The FC in the female gonad is plotted on the *X*-axis, and the FC in the male gonad is plotted on the *Y*-axis (Supplementary Figure S2). Genes that became enriched in testes relative to ovaries at T2 are shown in blue and are referred to as Male-enriched genes (MEGs). In contrast, genes that became enriched in ovaries relative to testes at T2 are shown in red and referred to as Female-enriched genes (FEGs). Genes that were similarly up- or downregulated in both sexes are shown in grey.

We pre-processed the data according to what is best for each type of source data (microarray or RNA-seq) and then we compared the resulting FC, which was calculated using exactly the same functions to build a linear model to determine DEGs between T1 and T2. The log_2_ transformed expression values at T2 were used to perform ANOVA analysis against values at T1 using the *limma* R package (Ritchie et al., 2015). The model was fit for all the probes using the *lmFit* function. The function *eBayes* was used to calculate moderated t-statistics and rank the statistical significance of each gene. Only significant genes (Benjamini–Hochberg adjusted *p* < 0.05) of FC higher than 1.5, or lower than −1.5, were kept for visualization of the results. Since RNA sequencing technology identifies more DEGs (Rao et al., 2019), we used more stringent filtering steps to make more comparable analysis between datasets obtained from microarray and RNA sequencing technologies. First, we removed genes considered as outliers when their read count was greater than 19.17 in Cooks distance between samples of the same group. Then, an independent filtering was also performed to optimize the multiple testing results with an alpha value of 0.1. Both the outlier removal and the independent filtering strategies were applied using the function *results* from the DESeq2 R package (Love et al., 2014; Love et al., 2022).

We generated a scatter plot for each species studied. Also, we produced line plots of expression profiles of few selected genes over time. Finally, we also created separated scatter plots of the FC for a panel of six key genes related to sex and reproduction: *sox9*, *dmrt1*, *amh*, Forkhead Box L2 (*foxl2*), gonadal aromatase (*cyp19a1*), and Follistatin (*fst*). Finally, we studied the mean FC and standard deviation of those genes considering the six species studied.

### 2.4 Gene and protein nomenclature

In this study we have used the existing and approved nomenclature, which was established in vertebrates 30 years ago and reflects evolutionary relationships (McCarthy et al., 2023). Thus, mammalian and avian gene symbols are in uppercase and italics (e.g., *AMH*), with the exception of rodents, which have gene symbols in lowercase and italics (e.g., *Amh*). Amphibian, reptile and fish gene symbols are in lower case and italics (e.g., *amh*). When referring to a gene in all vertebrates in general, gene symbols are in lower case and italics (e.g., *amh*). For proteins of vertebrates, we use the all uppercase gene symbol (e.g., AMH) as indicated in the International Protein Nomenclature Guidelines (NCBI, 2020).

### 2.5 Gene list enrichment analysis

We analyzed pathway enrichment for each species using the annotated FEGs and MEGs. The background gene set for each analysis consisted of genes captured and annotated in the respective microarray or RNA-sequencing datasets. We opted not to adjust *p*-values to avoid overly conservative results that might filter out interesting pathways. We used the PANTHER tool (Mi et al., 2019) with human genes as our reference database. We chose the human database because of its comprehensive annotation and completeness. This decision prevented bias towards any of the six selected species, ensuring fair comparisons. Our analysis considered both the percentage of genes annotated for each species and the uncorrected approach. Remarkably, in both cases, the top eight enriched and conserved pathways remained consistent across FEGs and MEGs.

## 3 Results

### 3.1 Sexually dimorphic gene expression patterns during development for each species

**FIGURE 1:**
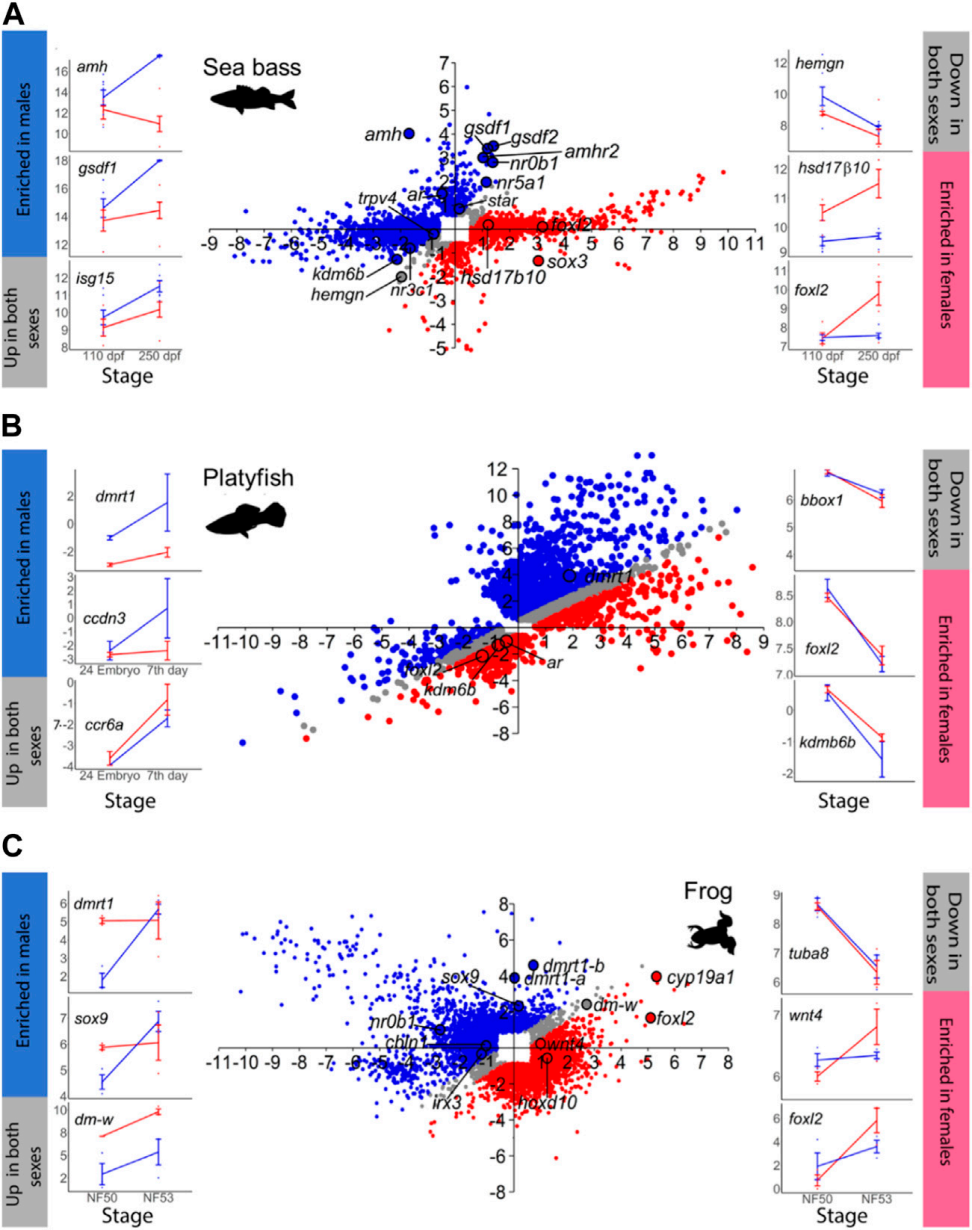
Gene expression changes at the beginning (T1) and towards the end (T2) of sexual differentiation in males and females. In the scatterplot central figures, axes indicate log_2_ FC. Probes that exhibited ≥1.5 FC and *p* < 0.05 in at least one sex between T1 and T2 are plotted in the *Y*-axis and in the *X*-axis representing testis and ovarian differentiation, respectively. Probes enriched in males at T2 are in blue, while probes enriched in females at T2 in red. Probes with similar expression in both sexes at T2 are plotted in grey. The line plots on the sides (blue lines = males; red line = females) show some examples of genes that are enriched in males (left, blue bar), that are upregulated in both sexes (left, grey bar), that are enriched in females (right, red bar), or that are downregulated in both sexes (right, grey bar). In the line plots, the *X*-axis indicates the developmental stage and the *Y*-axis represents log_2_ normalized counts per million (CPM) for RNA-seq-derived data or log_2_ normalized intensities for microarray-derived data. **(A)** Sea bass, **(B)** platyfish and **(C)** frog.

**FIGURE 2:**
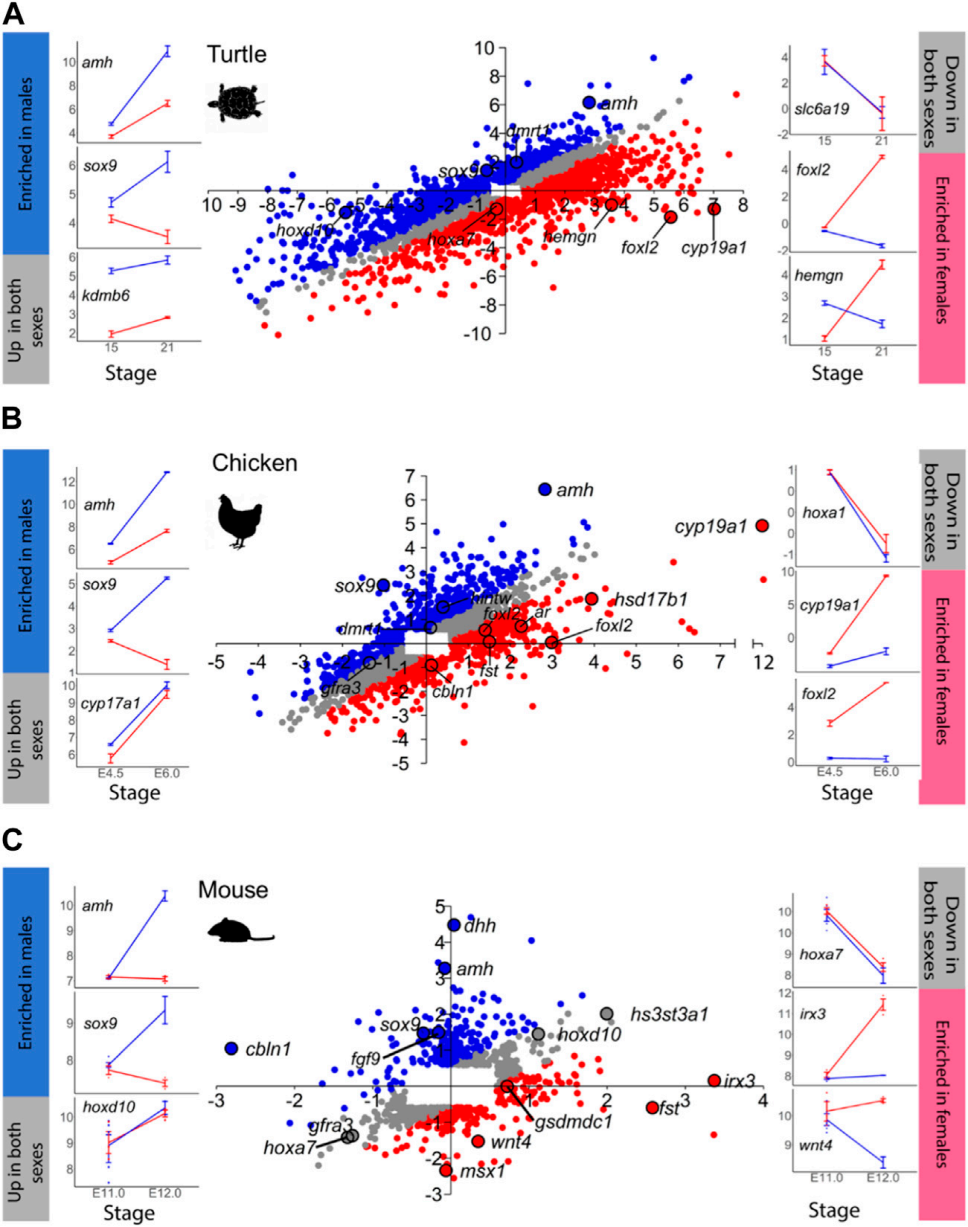
Same as in Figure 1 for: **(A)** turtle, **(B)** chicken, and **(C)** mouse. In C, the mouse scatterplot has been previously published by Munger et al. (2013).

The total number of MEGs and FEGs that were either upregulated or downregulated during ovarian differentiation and testicular differentiation varied greatly across the different vertebrate species analyzed (Table 1). Hence, sexually dimorphic gene expression in the gonad is achieved by activation and repression in different proportions across vertebrates (Figure 1; Figure 2). For example, in the European sea bass, there were a total of 4,129 DEGs out of the 20,978 annotated genes between 110 and 250 dpf in males and females (Figure 1A; Supplementary Table S2). Testis differentiation occurred through upregulation of 333 genes and concomitant downregulation of 254 genes. However, ovarian differentiation involved the upregulation of 1,605 genes combined with the downregulation of 1,646 genes. The remaining 292 DEGs between stages were regulated in the same direction in both sexes. In the platyfish, out of 24,209 annotated genes, there were 6,242 DEGs between 17 and 30 dpf (Figure 1B; Supplementary Table S3). Among the MEGs there were 1,652 genes upregulated in males and 198 downregulated in females while there were 340 FEGs upregulated in females and 1,039 FEGs downregulated in males.

**TABLE 1:** Differentially upregulated or downregulated genes between T1 and T2 of six species of vertebrates. The sex determining Mechanism (SDM), the method used to obtain the data (Microarray, M; RNA-sequencing, R) and the total number of DEG in each sex and the total number of coding genes present in each species are indicated. Other abbreviations: dpf, days-post fertilization; NF, Niewkoop and Faber; PSD, polygenic sex determination; TSD, temperature-dependent sex determination.

Group	Species	T1	T2	SDM	Method	References	Males		Females		Both sexes	Total DEGs	Total genes
							Up	Down	Up	Down			
Fish	European sea bass	110 dpf	250 dpf	PSD	M	Ribas et al. (2019)	333	254	1,605	1,646	292	4,129	20,978
	Platyfish	17 dpf	30 dpf	XY/XX	R	Present study	1,652	1,039	340	198	3,013	6,242	24,209
Amphibian	Frog	NF50	NF53	ZZ/ZW	M	Piprek et al. (2018)	738	1,309	1,098	1,753	536	5,434	20,219
Reptile	Turtle	15 stage	21 stage	TSD	R	Czerwinski et al. (2016)	371	441	1,037	876	1,605	4,330	28,415
Bird	Chicken	E.4.5	E6.0	ZZ/ZW	R	Ayers et al. (2015)	432	266	361	308	2,358	3,725	20,937
Mammalian	Mouse	E11.0	E12.0	XY/XX	M	Munger et al. (2013)	213	131	77	20	368	809	18,138

Another example is the red-eared slider turtle, which showed 4,330 DEGs between stages 15 and 21 out of the 28,415 annotated genes (Figure 2A; Supplementary Table S5). During testicular differentiation, 371 genes were upregulated in males while 876 genes were downregulated in females. Ovarian differentiation of the red-eared slider turtle involved the upregulation of 1,037 genes as well as the downregulation of 441 genes in males. A total of 1,605 genes appeared similarly regulated between stages 15 and 21 in males and females.

In the mouse (Figure 2C), we found 809 out of the 25,697 annotated genes that were differentially expressed between E.11.0 and E.12.0 in the 129S1 strain. Among the DEGs, 213 genes were upregulated in males and 20 were downregulated in females during testis differentiation. For development of the ovaries, 77 genes were upregulated in females combined with the downregulation of 131 genes in males (Figure 2C; Supplementary Table S7). These are exactly the same results described by Munger et al. (2013) when analyzing the mouse gonadal transcriptome. This is very important because: 1) it validates our analysis workflow and code to be used with the other species, and 2) allows meaningful comparisons across species since exactly the same method is applied. The total number of DEGs obtained for each species and sex is summarized in Table 1.

### 3.2 Global transcriptomic dynamics comparison across vertebrates

**FIGURE 3:**
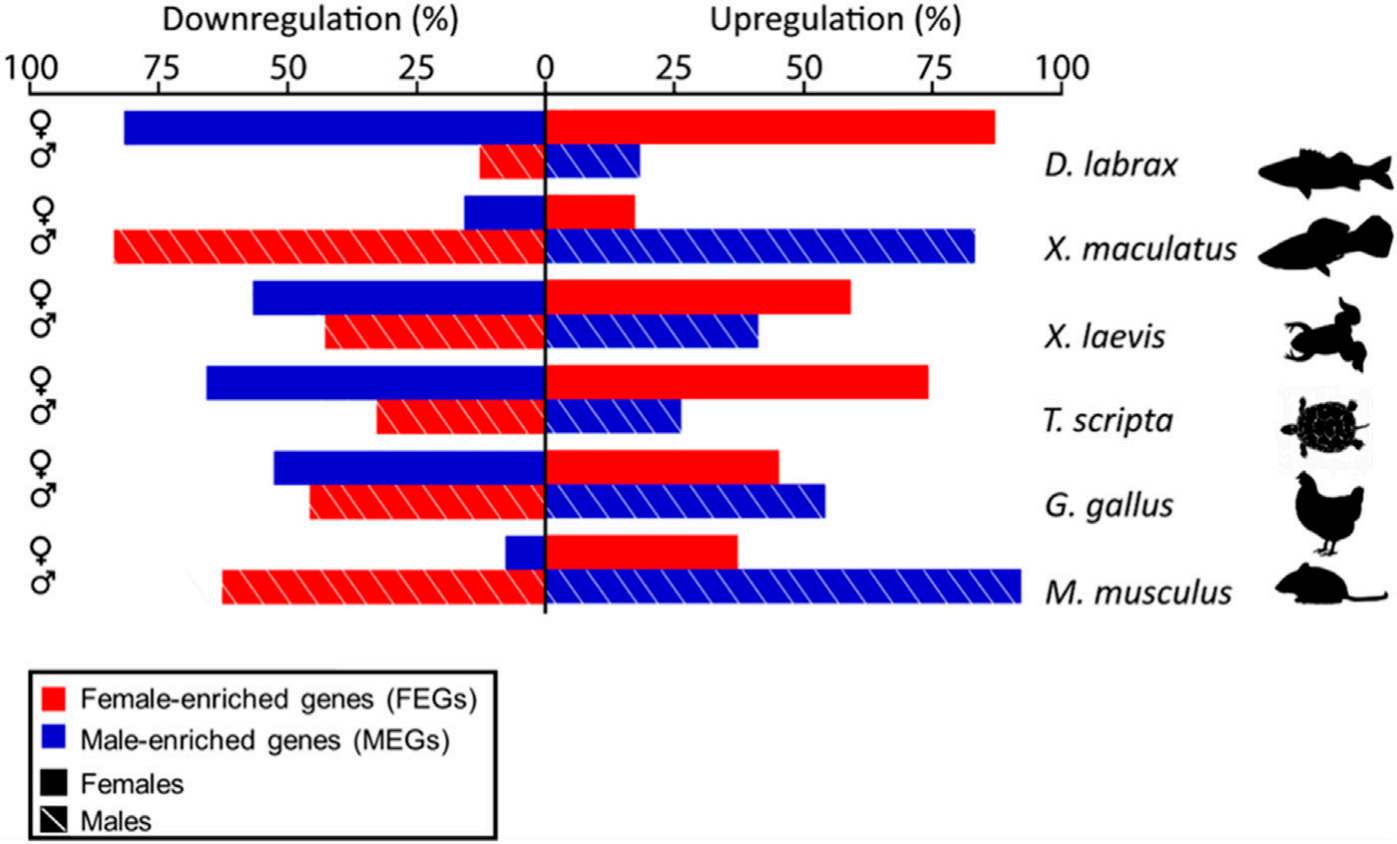
Bar plot showing the percent of male- (blue) or female-enriched (red) genes that are upregulated (right) or downregulated (left) during ovarian (top, solid lines) or testicular differentiation (bottom, stripped lines) in the different vertebrate species analyzed and compared in this study. Notice clear species- and sex-specific differences in the amount of activation and repression of male- and female-enriched genes.

Consistently across the species studied, higher expression in one sex was achieved not only through active upregulation in that sex but also by concomitant downregulation in the opposite sex or by a combination of both types of regulation. Moreover, important differences among species were evident. This can be clearly seen by plotting the percentage of upregulated and downregulated DEGs per sex in each species (Figure 3). Overall, we found that the ratio of MEGs and FEGs that were either upregulated or downregulated during ovarian and testicular differentiation greatly varied across the different vertebrate species analyzed, including the two fish species. For example, in the sea bass, ovarian development required upregulation of about 85% of FEGs and downregulation of a similar percentage of MEGs. In contrast, testicular differentiation involved a much smaller percentage of upregulated MEGs and downregulated FEGs, around 15%. In the platyfish, these percentages were essentially reversed and thus it was testicular rather than ovarian development that involved the major number of DEGs, both up- and downregulated. Similar to the platyfish, in the mouse, ovarian differentiation required a smaller percentage of upregulated FEGs (37%) or downregulated MEGs (8%) than testis differentiation (63% FEGs and 92% MEGs). Thus, in the sea bass ovarian differentiation requires simultaneous upregulation of most FEGs and concomitant downregulation of most MEGs, while in the platyfish and the mouse active up- and downregulation takes place during testis rather than in ovarian development. Finally, in the turtle and the frog the situation tended to resemble more the situation in the sea bass, albeit with less pronounced differences between the two sexes. The chicken, however, displayed the more balanced situation since the number of MEGs and FEGs upregulated and downregulated in each sex were similar.

### 3.3 Comparison of key gene dynamics across species

**FIGURE 4:**
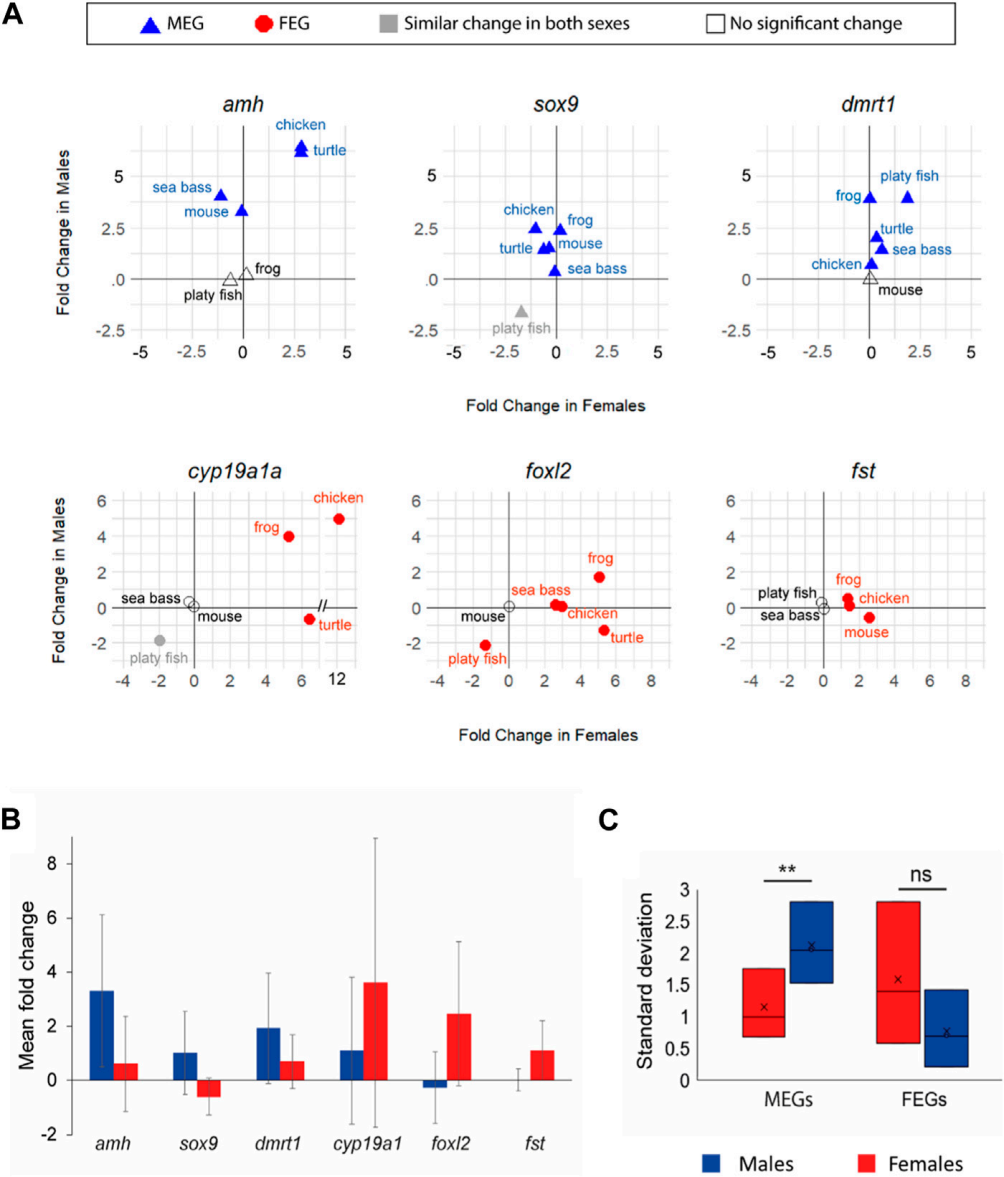
**(A)**. Expression changes (in log_2_ FC) of six key genes (*amh*, *sox9*, *dmrt1*, *cyp19a1a*, *foxl2*, and *fst*) involved in vertebrate sexual development between T1 and T2. Male-enriched genes (MEGs) at T2 are shown in blue triangles, Female-enriched genes (FEGs) at T2 are shown in red circles, genes without differences between sexes are plotted in grey, and genes without significant change are plotted with open symbols. **(B)** Bar plot of the mean FC of the six key genes (blue for males; red for females). Bars are standard deviation and indicate the variability of FC across species. **(C)** Box plot of the standard deviation of the same six key genes by sex (blue in males; red in females).

To directly examine the gene expression dynamics across species and by sex, we concentrated on a panel of previously described key genes. By ‘key genes’ we mean genes shown to be essential for sex differentiation in vertebrates (Ribas et al., 2017; Yildirim et al., 2020; Nagahama et al., 2021), although this knowledge has been driven by studies in mammals. From those genes we selected six for which a significant change in expression was found (FC ≥ 1.5 and *p* < 0.05) in at least three species and in at least one sex: *amh*, *sox9*, *dmrt1* in males and *cyp19a1a*, *foxl2*, and *fst* in females. We noted obvious differences across species (Figure 4A).

*amh* was clearly more enriched in males (FC > 5) with simultaneous gene expression increase in females (FC ∼2.5) in the chicken and turtle. In contrast, in the mouse upregulation occurred exclusively in males (X value ∼0). Yet, in the sea bass not only active upregulation in males but also concomitant downregulation in females was observed. In frog and the platyfish *amh* did not show any significant change, at least between the two developmental stages considered. The transcription factor *sox9* was consistently upregulated in males across species. However, it was downregulated during ovarian differentiation in the turtle, chicken and mouse but not in the frog and the sea bass. *dmrt1* was consistently upregulated in all species but the mouse, either only in males (frog and chicken) or also with concomitant upregulation in females (particularly in the platyfish, but also in the sea bass and turtle) albeit at lower FC values (Figure 4A).

Regarding the key FEGs, *cyp19a1a* was upregulated in both sexes but with higher FC values in females. In the turtle there was active downregulation in males whereas in the platyfish downregulation in both sexes was observed. The transcription factor *foxl2* showed the most variable dynamics across species. In the frog it was upregulated in both sexes, with a higher increase in females; upregulated in females without significant change in the males of the sea bass and the chicken, downregulated in both sexes (although enriched in females at the end of the studied period) of the platyfish, and it was upregulated in females while downregulated in males of the turtle. Lastly, *fst* was upregulated in both sexes, with a higher increase in females of the frog, upregulated in females of the chicken (no change in males), and upregulated in females while simultaneously being downregulated in males of the mouse (Figure 4A).

A particular case was that of the platyfish, species in which only two of the six key genes showed significant changes between stages: *foxl2* achieved female enrichment through downregulation in the males and *dmrt1* became a MEG through upregulation in the males. Collectively, these findings showed that among the six key genes selected *amh*, *sox9* and *dmrt1* were consistently MEGs, had similar change in both sexes or exhibited no significant change, but they never were FEGs. Conversely, *cyp19a1a*, *foxl2* and *fst* were consistently FEGs, had similar change in both sexes or exhibited no significant change, but they never were MEGs. This applied across the six studied species regardless of their sex determining system.

### 3.4 Variability of transcriptome dynamics of key genes of sexual differentiation across species

We also calculated the mean and standard deviation (SD) of the FC in males and females for each of the six key genes named above of the six studied species in order to better assess the magnitude and variability of the expression of these key genes according to sex. We observed higher mean FC and SD values in males for MEGs (*amh*, *sox9*, and *dmrt1*) and in females for FEGs (*cyp19a1*, *foxl2*, and *fst*) (Figure 4B). Furthermore, sex-related differences in SD values were significant (*p* < 0.01) for MEGs (Figure 4C). Together, these results suggest that the six selected key genes involved in testicular differentiation are mostly driven by upregulation while more variable dynamics were found for key FEGs, and that there is more gene expression variability across species in the sex for which their expression is most important (MEGs in males and FEGs in females).

### 3.5 Comparison of top gene dynamics across species

The approach followed in this study not only allows revealing important differences in the transcriptomic dynamics of key genes across species but also can help uncovering other important genes involved in sex differentiation of vertebrates previously not associated with this process. We reasoned that genes with the highest absolute FC values could be relevant. Thus, we filtered the datasets of the six species to retain only the top 10% of DEGs with highest absolute FC values regardless of sex and direction of change (from now on referred to as ‘top genes’). Then, we compared the six gene lists to find top genes shared among at least three or more species. There were 19 top genes including three key genes, i.e., genes previously known to have essential roles in sex differentiation of vertebrates: *amh*, *cyp19a1* and *foxl2* (Supplementary Table S7).

**FIGURE 5:**
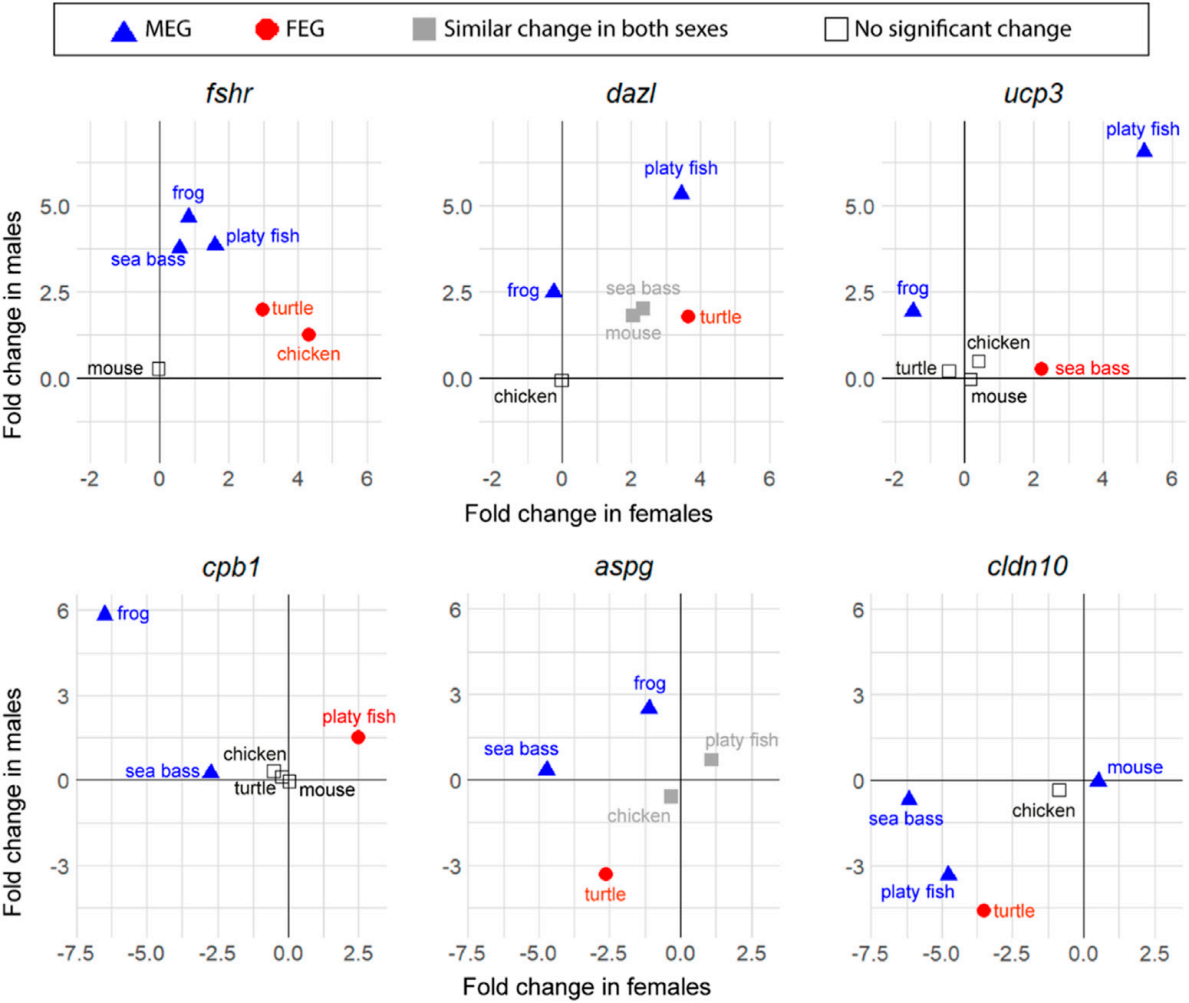
Changes in expression between T1 and T2 of six top genes involved in sexual development in vertebrates (in log_2_ FC): *fshr*, *dazl*, *ucp3*, *cpb1*, *aspg*, and *cldn10*. Male-enriched genes (MEGs) at T2 are shown in blue triangles, female-enriched genes (FEGs) at T2 are shown in red circles, genes with significant change between T1 and T2 but without differences between sexes at T2 are plotted in grey, and genes without significant change between T1 and T2 are plotted with open symbols.

Notably, while the six key genes were consistently enriched in one sex across all species compared (expression was either significantly enriched in one sex, in both, or non-significant), less conserved enrichment was found among the top genes—those that were not previously classified as key genes—which were enriched in one sex in some species and in the opposite sex in other species. Some examples are shown in Figure 5. Thus, in contrast to what has been shown earlier for the key genes, the most expressed genes are not necessarily enriched always in the same sex towards the end of sex differentiation in different vertebrate species.

The gene that stands out first is *fshr*. This gene is the receptor for follicle stimulating hormone and functions in gonad development of many species but is not always considered a key gene for vertebrates. The results show that even though it was not a DEG between stages in the mouse, important gene expression changes occurred in the rest of the five species studied. In the frog, the sea bass and the platyfish, *fshr* was classified as a MEG with relatively little change in the opposite sex, while in the turtle and the chicken, *fshr* was classified as a FEG, with moderate upregulation in males. This constitutes a clear example of genes enriched during sex differentiation in one sex or another depending on the species.

Some examples of other genes that met the strict filtering criteria to become top genes were: deleted in azoospermia like (*dazl*), an RNA binding protein classified as MEG in the platyfish and the frog but as FEG in the turtle. The gene uncoupling protein 3 (*ucp3*) encodes for a mitochondrial uncoupling protein member of the mitochondrial anion carrier proteins family. It was classified as a MEG in the platyfish and the frog but as a FEG in the sea bass. Carboxypeptidase B1 (*cpb1*) is an enzyme classified as MEG in the frog and the sea bass but as FEG in the platyfish. Asparaginase (*aspg*) is a coding enzyme protein classified as MEG in the frog and the sea bass but as FEG in the turtle. Claudin 10 (*cldn10*), which encodes for a component of the cellular membrane that works as a tight junction strand, is classified as MEG in the mouse, the platyfish and the sea bass but as FEG in the turtle.

**FIGURE 6:**
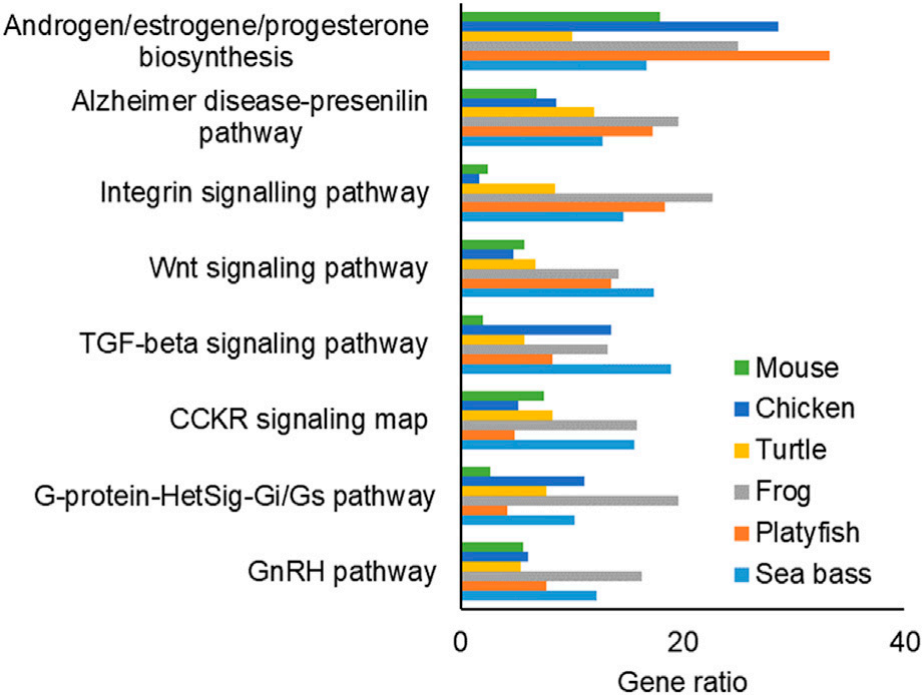
Bar plot of the most conserved and enriched pathways associated to the female enriched genes (FEGs). The *x*-axis indicates the gene ratio for each species. Abbreviations: TGF-beta: transforming growth factor-beta; CCKR: cholecystokinin receptor; G-protein-HetSig-Gi/Gs: Heterotrimeric G-protein Signal Transduction Pathway Involving Gi and Gs Proteins, GnRH: Gonadotropin-Releasing Hormone Signaling Pathway.

The pathway enrichment analysis of the FEGs (Figure 6) and MEGs (Supplementary Figure S3) resulted in 11 pathways which were the most enriched and most conserved patways across the studied species during sex differentiation. In addition, some pathways were enriched only in one species, such as the Endothelin signaling pathway (P00019), which was only enriched in FEGs of the mouse. However, we focused on those enriched and conserved pathways: among them, five were enriched in both FEGs and MEGs: Androgen/estrogen/progesterone biosynthesis, Alzheimer disease-prestilin pathway, Integrin signaling pathway, Transforming growth factor-beta signaling pathway and Gonadotropin-releasing hormone signaling pathway. Three conserved pathways were enriched by FEGs only: Wnt signaling pathway, cholecystokinin receptor signaling map, and Heterotrimeric G-protein signal transduction pathway involving Gi and Gs proteins pathway. Lastly, three conserved pathways were enriched by MEGs only: Adrenaline and noradrenaline biosynthesis, Oxidative stress response and Angiogenesis.

## 4 Discussion

Sex differentiation is a complex process orchestrated by numerous genes, tightly regulated spatially and temporally. The current consensus is that key genes involved in gonadal differentiation are relatively conserved, but that “their relative positions and, in some cases, their functions in testicular and ovarian differentiation differ” (Nagahama et al., 2021). This study delves into the true extent of conservation in the gene expression program involved in gonadal sex differentiation across species, at the beginning and end of this process. By conserved across species we mean: 1) genes expressed at comparable stages, 2) whether they participate in the differentiation of the same sex as inferred from a strong expression enrichment in that sex, and 3) what regulatory mechanisms (upregulation, downregulation or a combination of both) drive differential expression between the two sexes.

Traditionally, sex differentiation studies have focused on gene expression differences between one sex against the other. However, understanding this process also requires identifying mechanisms leading to gene enrichment in one sex relative to the other between two developmental stages, for each sex separately. Surprisingly, and to the best of our knowledge, this perspective has been explored only in mouse (Munger et al., 2013) and not in any other species. In this paper, we characterized expression dynamics in five other species, offering a systematic comparison across six vertebrates. We found that the six selected key genes of sex differentiation (*amh*, *sox9*, *dmrt1*, *cyp19a1*, *foxl2* and *fst*) show a consistent sex-specific expression trajectory connected to their known functions across the studied species (Nagahama et al., 2021). However, in this study, we also found that despite some players are fairly conserved, there are important overall differences in the expression dynamics between vertebrates. We hypothesize that the observed lack of conservation in sex differentiation mechanisms may be a strategy to ensure success of such an essential process: the development of ovaries and testis. While the final phenotypes remain invariable, likely due to purifying selection, those can be controlled by divergent genetic factors in different species as previously showed by some studies in which the molecular control and regulation of sex differentiation significantly differed even among closely related species with indistinguishable gonadal development at the morphological, histological and cellular levels (Herpin and Schartl, 2015; Capel, 2017; Cauret et al., 2020; Stöck et al., 2021). The great variability of dynamics and genes changing their function may allow for adjustments in a changing environment through speciation and evolution. Nevertheless, the dynamics and diversity of sex differentiation mechanisms remain underexplored and thus we encourage further research on more species, while highlighting the need to create new data of the gonadal transcriptome at comparable developmental stages.

Despite a careful selection of equivalent developmental stages was conducted, interpreting results requires caution, as alternate selection might yield different results. Comparing transcriptomic data across species poses challenges due to differences from adquisition methods. The comparison of data from microarray and RNA sequencing is possible, as high correlation between gene expression (FC) values was shown when relating the same samples in the two platforms (Black et al., 2014; Zhao et al., 2014). Nevertheless, several adjustments were made to ensure accurate data comparison (see methods section). Furthermore, we validated the results using raw data files from the mouse to exactly replicate the results through our pipeline. In comparing technologies, another potential concern is the absence of microarray probes for all genes. To mitigate this, we selected studies focused on gonadal development, ensuring the inclusion of crucial genes for gonadal development in each species and minimizing this issue. Additionally, we checked that the studies chosen capturated a relatively similar total number of genes (Table 1). Yet, the common limitation of all transcriptomic comparative studies is that these directly rely on the quality of reference genome annotation (Roux et al., 2015).

Despite these limitations, our results disclose new insights for the understanding of the complex process of sex differentiation in vertebrates. Overall, we found that a large proportion, between 3% and 27% (mean = 18%), of the known coding genes changed their expression between T1 and T2 in all studied species. The effective manner to display gonadal transcriptomic data designed by Munger et al. (2013) in a scatterplot allowed obtaining a global view of the mechanisms used to achieve dimorphic expression changes in the gonad of the mouse. Surprisingly, such proportions remained unknown in other vertebrates until now. The present study reveals that these proportions are completely different in other species. To date, the only study that globally compared transcriptomes during sexual differentiation was focused on studying the order in which groups of genes were expressed simultaneously or not between turtle and mouse (Czerwinski et al., 2016). These authors found that even though antagonistic forces were conserved, groups of shared genes between the mouse and the turtle showed heterochronic dynamics of genes which converged to upregulate or downregulate aromatase (Czerwinski et al., 2016).

Using such approach of the scatterplot in the mouse, it was evident that “downregulation in the opposite sex” is also required to reach gene dimorphism. In mouse, testis development involves changes (up- and downregulation) of a greater number of genes than ovarian differentiation. This observation aligns with the concept proposed by Jost (1947) regarding sex differentiation stating that females are the default sex, less dependent on gonadal hormones. Regarding the other species, we found for the first time that the same mechanism also occurs in the platyfish by a different proportion of genes (about 85% of the FEGs achieved enrichment through downregulation in the males). Surprisingly, the opposite strategy occurs in other species: a large proportion of genes reached higher expression in males through downregulation in females. This was found in the sea bass (82% of its MEGs) and, to a lesser extent, in the turtle (66%) and the frog (57%). Hence, a higher number of genes required a change of expression to develop an ovary than to develop a testis. Taken together, these results reveal that there is no relationship between how dimorphic expression in the gonad is achieved and position in the vertebrate phylogenetic tree.

The comparison of the gonadal transcriptome between species confirms that the key genes for sex differentiation that we studied are fairly conserved, as previously suggested (Herpin and Schartl, 2015; Capel, 2017; Nagahama et al., 2021). For example, *amh*, *sox9* and *dmrt1* achieved sex-dimorphic expression through upregulation in males and relatively little or no change in females. Interestingly, the standard deviation of the mean FC of these genes between species was higher in the males than in the females, suggesting that evolutionary pressures could be acting in a sex-specific manner. On the other hand, key genes related to ovarian development (*cyp19a1*, *foxl2* and *fst*) showed more variable expression dynamics. Such type of information was, to the best of our knowledge, not shown in such a clear and comparative manner as the present study does. In other cases, genes such as the androgen receptor (*ar*), showed less degree of conservation across species regarding its expression during gonadal differentiation. Thus, these results support the notion that important genes composing the network involved in differentiation can be substantially different even among closely-related species (Herpin and Schartl, 2015; Capel, 2017).

Then, we wondered whether the approach used in this study could also reveal genes that had not been considered as key genes in gonad sex differentiation. We sought to determine if this approach could reveal genes not traditionally associated with sex differentiation in gonads. Thus, we searched among the top 10% genes with the highest FC between T1 and T2, in an analogous manner than in studies seeking gene markers (Miller et al., 2011; Marshall et al., 2013; Khazaei et al., 2018). We refer to these genes as ‘top genes.' Among these top genes, we found some previously unrelated to gonadal development, such as *myl2*, involved in embryonic heart muscle function (Weterman et al., 2013). Many top genes were known for roles in reproduction or gonadal expression but they were not previously associated to differentiation. For instance, *dazl*, involved in differentiation of germ cells (Yu et al., 2009); *cldn10*, an ovarian cancer biomarker in the chicken (Seo et al., 2010); and *asz1*, associated with gametogenesis and piRNA metabolism (Yi et al., 2014; Wang et al., 2017).

Among the top genes identified, *fshr* deserves attention. Although it was not enriched in the same sex for all species, high significant expression changes were found in sea bass, platyfish, and frog as MEG, and in turtle and chicken as FEGs. *fshr* is a an important gene for gonadal development acting downstream of the pituitary-secreted FSH from fish to mammals (Nakamura, 2013; Akazome et al., 2002; White and Thomas, 1992; Grzegorzewska et al., 2009; Hollander-Cohen et al., 2021). Interestigly, Hollander-Cohen et al. (2021) found that across vertebrates, there are two types of gonadotrophic cells: bi-hormonal (1 cell secreting both LH and FSH), evolved in amphibians, reptiles, and mammals; and mono-hormonal cells (two different cells secreting each LH or FSH), in teleosts and avian. This is thought to be a case of convergent evolution. In our results, *fshr* was FEG in the chicken but MEG in the two species of teleosts considered, which would be in accordance of convergent evolution through different regulatory mechanisms. However, how this unique change in the cell morphology contributes to the reproductive strategy, functionality and downstream regulation of sex developmet is not known. Thus, we suggest that this gene should be considered as a key gene involved in vertebrate gonadal sex differentiation.

In this study, we identified several enriched and highly conserved pathways associated with FEGs and MEGs that have known roles in gonadal differentiation. These pathways include Androgen/estrogen/progesterone biosynthesis, Gonadotropin-releasing hormone signaling, and TGF-beta signaling patways. Previous research has established their importance in the development of both ovaries and testes in vertebrates (Haffen et al., 1970; Kavanaugh et al., 2008; Wen et al., 2010; Bondesson et al., 2015; Zheng et al., 2018; Pan et al., 2021). Similarly, our findings also reaffirmed the significance of the Wnt signaling pathway in vertebrate ovarian development (Chassot et al., 2014; Sreenivasan et al., 2014). In addition to these well-known pathways, we identified other highly enriched and conserved pathways that have received less attention in the literature. Notably, the Integrin signaling pathway and the Alzheimer’s disease-presenilin pathway fall into this category.

One study by Bhandari et al. (2020), supports the potential link between the Integrin signaling pathway and the gonad’s steroid-producing capacity. Enrichment of the Integrin signaling pathway was found in the testes of medaka fish (*Oryzias latipes*) exposed to low concentrations of Bisphenol A (BPA) and 17α-ethinylestradiol (EE2), two common endocrine-disrupting chemicals in the environment. Similarly, the Alzheimer’s disease-presenilin pathway was enriched in the testes of medaka exposed to EE2. While mutations in presenilin proteins are known to cause Alzheimer’s disease in brain cells, the role of genes in this pathway can differ when expressed in different organs and developmental stages. Taken together, the comparative analysis of enriched pathways highlighted both well-established and lesser-known pathways involved in gonadal differentiation of vertebrates. Further research into these pathways and their specific roles in gonadal development can provide valuable insights into its evolution.

The evolution of genes and mechanisms involved in vertebrate sexual development has given rise to various theories, including the bottom-up theory proposed by Wilkins (1995). This theory suggests that purifying selection acts only at the bottom of a hierarchical cascade. This theory was supported by Graham et al. (2003) who referred to the genes at the top as “masters" and those at the bottom as “slaves". Accordingly, while masters can vary from one species to another, the slaves maintain structural and functional conservation across species (Graham et al., 2003; Williams and Carroll, 2009). Since then, subsequent studies revealed a more complex network of antagonizing pathways involved in gonadal sex differentiation and, importantly, with different genes participating in the regulation of male and female pathways even between closely related species (Heule et al., 2014; Herpin and Schartl, 2015; Pan et al., 2016; Stöck et al., 2021; Martinez-et al., 2022). Nowadays it is accepted that, despite the diversity of sex determining mechanisms, gonadal development involves a complex network of multiple regulatory interactions of genes associated with male and female pathways. Those are expressed simultaneously as opposing forces, until a factor or combination of them tilts the balance towards one fate or the other (Capel, 2017). Our results support this view and provide the first truly comprehensive and quantitative examination of gene expression differences during sex differentiation across vertebrates.

As shown in this study, the consistent enrichment of the six selected key genes for gonadal sex differentiation exclusively in one sex aligns with Lynch’s hypothesis. Lynch hypothesis states that only the final gene product produces a phenotype exposed to selection (Lynch, 2007), allowing developmental pressures to act upstream of the key genes without changing the final phenotype (Lynch, 2007; Herpin and Schartl, 2015). However, genes such as *fshr* and *ar* were involved in testicular or ovarian differentiation depending on the species, supporting the hypothesis based on the link between a sex-determining gene to a sex differentiation gene (van Doorn and Kirkpatrick, 2007; Herpin and Schartl, 2015). Considering the behavior of key genes (if enriched, always in the same sex) and the top genes (enriched in one sex or the other, depending on the species), it is tempting to propose that a hierarchical network with important hub genes and less connected nodes control the process rather than a hierarchical cascade nor a plain network. Hub genes would correspond to genes, including key genes, consistently enriched in the same sex while less connected genes would be those genes involved in testicular or ovarian differentiation depending on the species. Networks with hub genes, some of them key genes such as *sox9* and *amh*, were characterized during gonadal sex differentiation of sea bass, mouse and humans (Sánchez-Baizán et al., 2022). Thus, in such a scenario, one could speculate that evolutionary pressures may affect genes depending on the number of connections in the network, with hub genes more robust to changes, which would explain why, if they are enriched, they are always enriched in the same sex at the end of gonadal differentiation across vertebrates. This is compatible with the basic underlying principle about the existence of antagonizing signals that ensure canalization of the male or female developmental pathway and that tolerate plasticity (Capel, 2017).

To conclude, we investigated the variability in gene expression dynamics during gonadal sex differentiation across six vertebrate species. The proportion of genes being up- or downregulated leading to sex-related differences in gene expression varied greatly across species. Interestingly, a large proportion of genes acquired enrichment through downregulation in the opposite sex, not only in the mouse but also in other vertebrates. Also, the six studied key genes were consistently enriched in one sex only (if enriched) across the six studied species. Among these key genes, those involved in testicular differentiation were mostly driven by upregulation, while key genes associated with ovarian differentiation exhibited more varied dynamics. Additionally, we identified 16 new markers of early sex differentiation in vertebrates and identified 11 enriched pathways associated to FEGs and MEGs. Based on the results of this study and previous literature we suggest that a hierarchical network with hub genes and less connected nodes underlies the process of sex differentiation, influenced by evolutionary pressures that depend on the number of gene connections. These findings could have important implications for understanding the evolution of sex determination and differentiation in vertebrates.

## Data Availability

The datasets presented in this study can be found in online repositories. The names of the repository/repositories and accession number(s) can be found in the article/Supplementary Material.
