# Supplementary material for: Extraordinary variability in gene activation and repression programs during gonadal sex differentiation across vertebrates
Source: Front Cell Dev Biol. 2024 Jan 23;12:1328365. doi: 10.3389/fcell.2024.1328365 (PMC10844511; doi:10.3389/fcell.2024.1328365)
Supplement: Supplementary file 1 [file DataSheet1.PDF]

Supplementary Information

Extraordinary variability in gene activation and repression programs during gonadal sex differentiation across vertebrates

Núria Sánchez-Baizán[1,], Ignasi Jarne-Sanz[1], Alvaro S. Roco[2,3], Manfred Schartl[2,4], Francesc Piferrer[1],*

1. Institut de Ciències del Mar (ICM), Spanish National Research Council (CSIC), Barcelona, Spain
2. Developmental Biochemistry, Biocenter, University of Wuerzburg, 97074 Wuerzburg, Germany
3. Department of Experimental Biology, Faculty of Experimental Sciences, University of Jaén, Campus Las Lagunillas S/N, 23071 Jaén, Spain
4. Xiphophorus Genetic Stock Center, Texas State University, San Marcos, TX 78666, USA

*Correspondence: Dr. Francesc Piferrer, Institut de Ciències del Mar (ICM), Spanish National Research Council (CSIC), Passeig Marítim, 37-45, 08003 Barcelona, Spain. Tel. +34-932309567.

E-mail: piferrer@icm.csic.es

Background information of the selected species

Fishes

Teleosts are the vertebrates exhibiting the highest variety of sexual systems, where gonochorism, hermaphroditism (both simultaneous and sequential) and unisexuality are found. However, about 95% of teleosts are gonochoristic (Pla et al., 2021). Also, they are the group with the most varied range of sex determination mechanisms from GSD to ESD, including chromosomal sex determination (CSD) and polygenic sex determination (PSD) (Mank et al., 2006; Schartl et al., 2023).

The sea bass (*Dicentrarchus labrax*) is a gonochoristic species with PSD influenced by the environment (Piferrer et al., 2005; Vandeputte et al., 2007; Vandeputte and Piferrer, 2019). The first signs of gonadal differentiation at the molecular level are found at 110 days post fertilization (dpf), where the expression of several genes becomes sexually dimorphic: for example the markers: Hydroxysteroid 17-Beta Dehydrogenase 10 (*hsd17β10*), upregulated in females (Ribas et al., 2019) and *sox9a* upregulated in males (Sánchez-Baizán et al., 2022). Nevertheless, the first marker identified of ovarian differentiation is gonadal aromatase, Cytochrome P450 Family 19 Subfamily A Member 1 (*cyp19a1a*), which is expressed higher in females around 120 dpf (Blázquez et al., 2008). Sex differentiation is complete by 200 dpf. The gonads used in this study were from fish of 5.2 ± 0.5 cm of standard length (SD) at 110 dpf, females of 12.7± 5.7 cm of SD at 250 dpf and males of 11.2 ± 0.6 cm of SD at 250 dpf.

The platyfish (*Xiphophorus maculatus*), is a species of the livebearing (ovoviviparous) genus *Xiphophorus*. This genus is particularly diverse in sex-determining systems. The platyfish has a CSD with multiple sex chromosome mechanisms (WY, WX or XX females; XY or YY males) (Kallman, 1984; Volff and Schartl, 2002)(Zhang et al., 2011). Although the gonadal transcriptome has been studied to identify genes differentially expressed between male and female gonads in adults, the transcriptome has never been studied during the period of sex differentiation. RNA-seq transcriptomes were obtained from T1 = embryonic stage 24 (approx. 17 dpf, (Tavolga, 1949) which follows the sex determination stage, and T2 = 7 d old newborn fish (approx. 30 dpf), representing the later gonadal differentiation stage. The

highly inbred platyfish strain Jp163A (WLC#6628) was used, which has XY sex determination with no environmental modification noticed under laboratory conditions. All animals were kept and sampled in accordance with the applicable EU and national German legislation governing animal experimentation. In particular, all experimental protocols were approved through an authorization (55.2532-2-215) of the Veterinary Office of the District Government of Lower Franconia, Germany, in accordance with the German Animal Protection Law (TierSchG) and in accordance with ARRIVE guidelines. All fish were cut into three pieces: head, abdomen with gut removed, and tail. The head and tail portion were used for PCR genotyping and the abdomen containing the developing gonad for RNA extraction.

Amphibians

Amphibians exhibit GSD with either male (XY/XX) or female (ZZ/ZW) heterogamety. The only sex-determining gene characterized in anurans so far is the W-linked DM-domain gene (*dm-w*). *dm-w* is a female-specific gene and was described in the African clawed frog (*Xenopus laevis*) with ZZ/ZW heterogamety (Yoshimoto et al., 2008). However, this gene did not show a relevant role during gonad differentiation (Nagahama et al., 2021; Stöck et al., 2021). Instead, key genes involved in ovarian differentiation of *X. laevis* are *foxl2*, *cyp19a1*, and *fst*. The development of testis characterized by the expression of *amh*, Cytochrome P450 Family 17 Subfamily A Member 1 (*cyp17a1*), *dmrt1*, Follicle Stimulating Hormone Receptor (*fshr*), *ptgds*, and *sox9* (Piprek et al., 2018). The individuals used in this study were staged according to Nieuwkoop and Faber (1964) and the selected stages were NF50 and NF53. Morphologically, at NF50 the gonads are undifferentiated with few germs cells. The earliest visible signs of differentiation were described at NF53 (approximately 23 dpf) when there is visible difference in the location of the germ cells (Piprek et al., 2017). However, at the molecular level there were clear changes of gene expression of sex differentiation key genes (Piprek et al., 2018). We highlight that (Piprek et al., 2017) defined NF50 as undifferentiated gonads and the NF53 stage as the onset of differentiation based on morphological differences. However, at the molecular level the differences appeared prior to morphological differentiation. We selected the chosen stages based on several comparisons of the transcriptome between NF50, NF53 and NF56 as well as the results from Piprek et al.,

(2018) to finally consider NF50 as the beginning of molecular differentiation and NF53 towards the end because at NF56 the ovaries are completely recognizable.

Reptiles

Most squamates present GSD while ESD is predominant in all crocodiles, most turtles, and some lizards. To represent reptiles, we studied the gonadal transcriptome of a turtle (*Trachemys scripta elegans*). This species is a TSD species, whose sex differentiation starts at stage 15 and ends at stage 21. The male gonadal development begins with the activation of *dmrt1*,which leads to upregulation of *amh* and *sox9*. At female promoting temperatures (FPT), *cyp19a1* and *foxl2* are the key genes leading to ovarian differentiation (Czerwinski et al., 2016; Nagahama et al., 2021). The data used in this study was obtained from gonads sampled from embryos kept at male- or female-producing temperatures. The stages selected for the study were stage 15 and stage 21 based on the comprehensive time course analysis of gonadal development. Czerwinski et al., (2016) found only a total of 10 DEGs between sexes at stage 12, and hence, considered the beginning of molecular sex differentiation at stage 15. At stage 21 the gonadal phenotype is irrevocably set and towards the end of molecular changes to achieve differential expression between sexes.

Birds

Birds evolved from theropod dinosaurs in the late Jurassic and, similar to some amphibians, some reptiles and numerous fish species, they have female-heterogametic sex chromosomes, i.e., a ZZ/ZW system (Fridolfsson et al., 1998). Yet, unlike in *Xenopus*, a female sex-determining gene has not been discovered. Instead, testis development is initiated by the activity of the double doses of *DMRT1* in the primordial germ cells, followed by coordinated activation of *HEMGN*, *AMH*, and *SOX9*. In contrast, ovarian development is dominated by estrogen production regulated through genes such as *CYP19A1*, *FOXL2*, R-Spondin 1 (*RSPO1*), and *WNT4* (Chue and Smith, 2011; Nagahama et al., 2021). The samples used were studied by (Ayers et al., 2015) who compared embryonic stages E4.5 (stage 26) and E6.0 (stage 29; (Hamburger and Hamilton, 1951) stages). They classified gonads at E4.5 at the beginning of differentiation or “prior to differentiation” and differentiating gonads at E6.0.

Mammals

Placental mammals have GSD with heteromorphic sexual chromosomes (XX/XY) system., The mouse, *Mus musculus*, is the model organism for which sexual development is most well-known within vertebrates. Up to the embryonic stage (E.)11.2 the genital ridges are bipotential. The *Sry* determining gene is found in the Y chromosome and initiates the development of testes between E.10.0 and E.11.2. *Sry*, activates *Sox9* with a positive feedback loop existing between *Pdg2*/*Sox9* and Fibroblast Growth Factor 9 (*Fgf9*)/*Sox9*. In the XX gonads, genes such as *Rspo1*, *Wnt4*, and *Foxl2* are expressed during early ovarian development (Wang et al., 2019; Yildirim et al., 2020). The data used for the present study were obtained from Munger et al., (2013) who sampled individually staged gonads by the number of somites of the embryos. The selected stages were from individuals at E11.0 and E12.0 of the 129S1 strain.

Supplementary figures

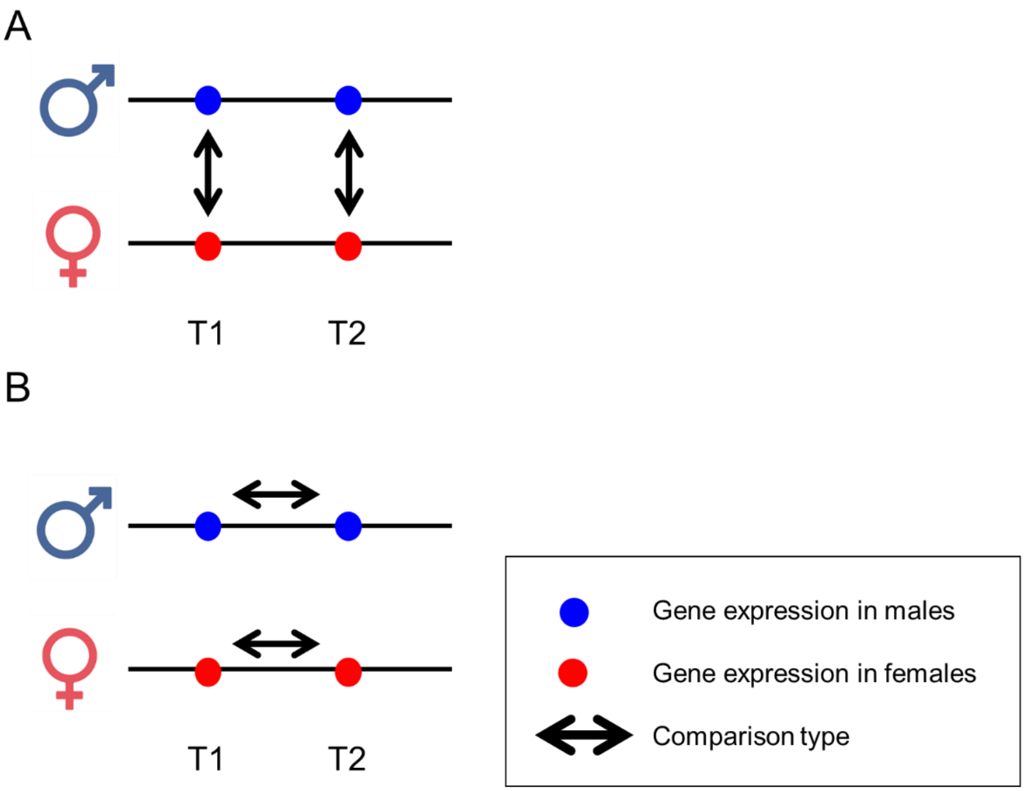

Supplementary figure 1. Diagram of the two comparisons discussed in the present study. **A**. The gonadal transcriptomes of the two sexes are compared at one or more developmental times (T1, T2) or stages. **B**. The gonadal transcriptome at two different developmental times or stages, e.g., at the beginning (T1) and towards the end (T2) of the sex differentiation process are compared within each sex.

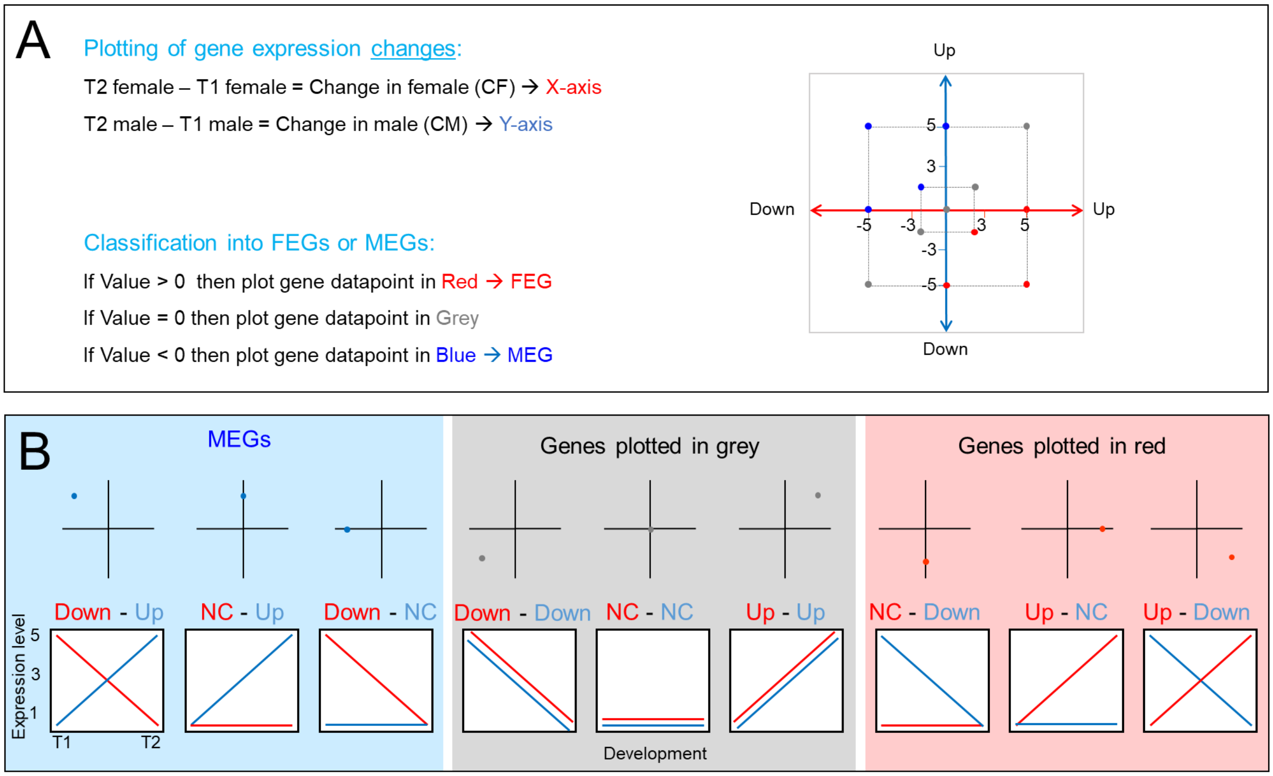

Supplementary figure 2. Diagram of the approach used to study transcriptomic dynamics using the "horizontal comparison", T2 vs T1, as described in Suppl. Fig. 1B. The two gene expression data points or stages (T1 and T2 in males and females) make up a total of four data points that are converted into a single data point in the X-Y coordinates of a scatterplot, in units of fold change (FC). Abbreviations: MEGs, male-enriched genes; FEGs, female-enriched genes. **A.** The diagram shows where the FC is placed for each sex. Also, it indicates the directions of the regulatory mechanisms (up- and downregulation for each sex, i.e., each axis). **B**. The nine major theoretical possible combinations of regulatory mechanisms for males (blue line) and females (red line) between two developmental time points or stages (T1 and T2) using theoretical gene expression values (0 to 5). These are: 1. Down in female and up in male, 2. No change in female and up in male, 3. Down in female and no change in male, 4. Down in both sexes, 5. No change in both sexes, 6. Up in both sexes, 7. No change in females and down in males, 8. Up in females and no change in males, 9. Up in females and down in males. The cross above represents the X and Y axes shown in A and the dot the corresponding data point. The blue background depicts instances where a given gene is classified as a MEG, and this can be achieved basically in three ways between T1 and T2: 1) upregulation in males and concomitant downregulation in females (left panel), 2) upregulation in males and no change in females (middle panel), and 3) no change in males and downregulation in females (right panel). The same principle but with opposite direction changes apply for the FEG (red background).

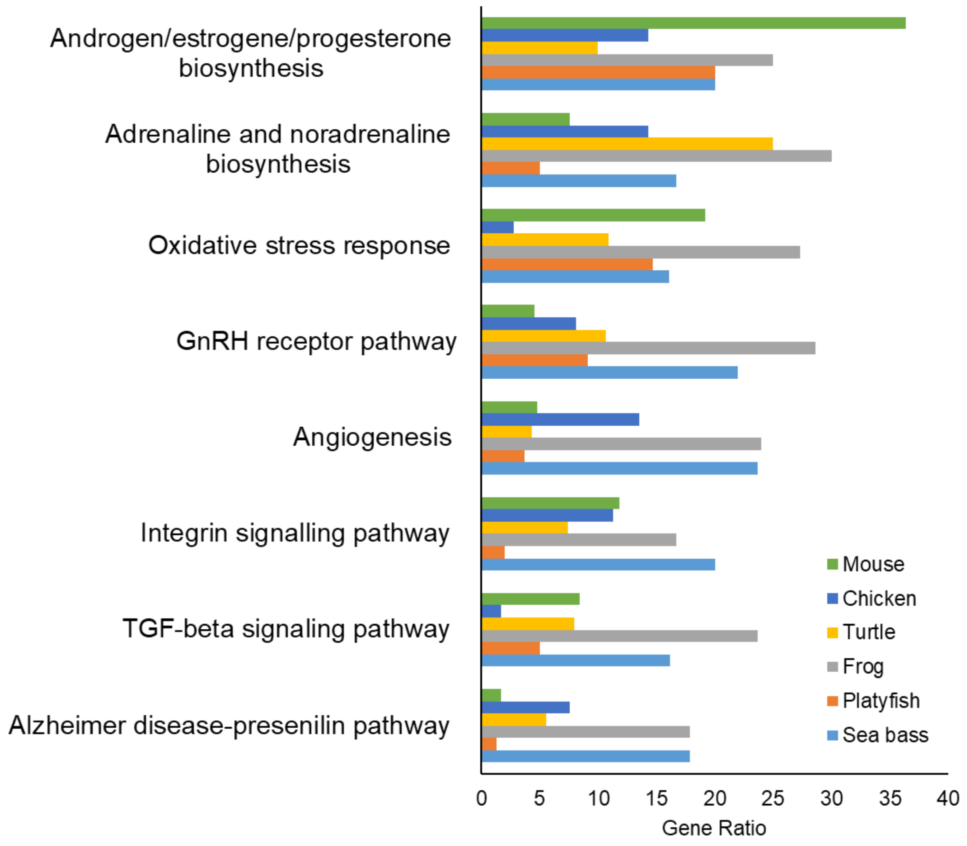

Supplementary figure 3. Bar plot of the most conserved and enriched pathways associated to the male enriched genes (MEGs). The x-axis indicates the gene ratio for each species. Abbreviations: GnRH: Gonadotropin-Releasing Hormone Signaling Pathway, TGF-beta: transforming growth factor-beta.

Supplementary tables

Supplementary tables 1 through 6 are contained in an additional Excel file

Supplementary table 1. Fishes. Differentially expressed genes (DEGs) between 110 and 250 days post fertilization (dpf) in males and females of the European sea bass. The table includes information on gene ID and gene description. It also includes the following information for each sex: $\log_2$ fold change between stages (logFC), average expression (AveExpr), t statistic value (t), *P* - value (P.value), and adjusted *P* – value (adj.P.val). Information for each sex is indicated with .x for data from the female samples and .y for data from the male samples.

Supplementary table 2. Fishes. DEGs between 17 and 30 dpf in males and females of the platyfish. The table includes information on gene ID and gene description. It also includes the following information for each sex: $\log_2$ fold change between stages (logFC), average expression (AveExpr), t statistic value (t), *P* - value (P.value), and adjusted *P* – value (adj.P.val). Information for each sex is indicated with .x for data from the female samples and .y for data from the male samples.

Supplementary table 3. Amphibians. DEGs between stages NF50 and NF53 in males and females of the African clawed frog. The table includes information on gene ID and gene description. It also includes the following information for each sex: $\log_2$ fold change between stages (logFC), average expression (AveExpr), t statistic value (t), *P* - value (P.value), and adjusted *P* – value (adj.P.val). Information for each sex is indicated with .x for data from the female samples and .y for data from the male samples.

Supplementary table 4. Reptiles. DEGs between 15 and 21 days of development in males and females of the red-eared slider turtle. The table includes information on gene ID and gene description. It also includes the following information for each sex: : $\log_2$ fold change between stages (logFC), average expression (AveExpr), t statistic value (t), *P* - value (P.value), and adjusted *P* – value (adj.P.val). Information for each sex is indicated with .x for data from the female samples and .y for data from the male samples.

Supplementary table 5. Birds. DEGs between stages E.4.5 and E.6.0 in males and females of the chicken. The table includes information on gene ID and gene description. It also includes the following information for each sex: $\log_2$ fold change between stages (logFC), average expression (AveExpr), t statistic value (t), *P* - value (P.value), and adjusted *P* – value (adj.P.val). Information for each sex is indicated with .x for data from the female samples and .y for data from the male samples.

Supplementary table 6. Mammals. DEGs between stages E.11.0 and E.12.0 in males and females of the mouse. The table includes information on gene ID and gene description. It also includes the following information for each sex: $\log_2$ fold change between stages (logFC), average expression (AveExpr), t statistic value (t), *P* - value (P.value), and adjusted *P* – value (adj.P.val). Information for each sex is indicated with .x for data from the female samples and .y for data from the male samples.

Supplementary table 7. List of the genes found in common in at least three of the six species of vertebrates considered in this study which were amongst the top 10%-fold change expression between stages in at least one sex, either up or downregulated. Numbers in parenthesis indicate the number of species. Among these 19 genes there are three (highlighted in bold) previously known to be involved in sex differentiation (named as key genes in this study). The information in the 'Gene type' column was obtained from GeneCards database (Stelzer et al., 2016)

Species where found as top genes	Gene symbol	Gene name	Gene type
(4) sea bass, frog, turtle, chicken	***foxl2***	Forkhead Box L2	Transcription factor
(4) sea bass, frog, turtle and chicken	*fshr*	Follicle Stimulating Hormone Receptor	Receptor
(4) sea bass, turtle, chicken, mouse	***amh***	Anti-Müllerian Hormone	Protein hormone
(3) frog, turtle, chicken	***cyp19a1***	Cytochrome P450 Family 19 Subfamily A Member 1	Enzyme
(3) platyfish, sea bass, frog	*asz1*	Ankyrin Repeat, SAM And Basic Leucine Zipper Domain Containing 1	Binding protein
	cpb1	Carboxypeptidase B1	Enzyme
	ucp3	Uncoupling Protein 3	Mitochondrial uncoupling protein
(3) sea bass, frog, chicken	*myl2*	Myosin Light Chain 2	Sarcomeric protein
	iyd	Iodotyrosine Deiodinase	Enzyme
(3) frog, chicken, mouse	*spon2*	Spondin2	Binding protein
(3) sea bass, frog, mouse	*eno3*	Enolase3	Isoenzyme
(3) sea bass, frog, turtle	*hmg20a*	High Mobility Group 20A	Binding protein
	aspg	Asparaginase	Enzyme
(3) sea bass, turtle, chicken	*g0s2*	G0/G1 Switch 2	Mitochondrial protein
(3) platyfish, frog, chicken	*unc45b*	Unc-45 Myosin Chaperone B	Regulatory component of progesterone receptor
	ccna1	Cyclin A1	Binding protein
(3) platyfish, frog, turtle	*dazl*	Deleted In Azoospermia Like	RNA binding protein
(3) platyfish, turtle, mouse	*cdkn1a*	Cyclin Dependent Kinase Inhibitor 1A	Inhibitor enzyme
(3) platyfish, sea bass, turtle	*cldn10*	Claudin 10	Tight junction strand (integral membrane protein)
